# Applying a Pharmacometrics‐Enabled Machine Learning Analysis to Predict 2‐Month Culture Conversion Using Phase 2a Data in a Tuberculosis Clinical Trial

**DOI:** 10.1111/cts.70694

**Published:** 2026-08-10

**Authors:** Huifang You, Ulrika S. H. Simonsson

**Affiliations:** ^1^ Department of Pharmaceutical Biosciences Uppsala University Uppsala Sweden

**Keywords:** early bactericidal activity, machine learning, phase 2a, phase 2b, TTP, tuberculosis

## Abstract

Phase 2a trials in tuberculosis patients traditionally assess early bactericidal activity over two weeks, often using the time‐to‐positivity biomarker, followed by a phase 2b study typically lasting 8‐week with time‐to‐event of culture conversion as the endpoint. This study investigated different machine learning models to predict the time‐to‐event of 2‐month culture conversion in the REMoxTB trial with phase 2a time‐to‐positivity biomarker data and the impact of different phase 2a study lengths. Time‐to‐positivity at baseline and up to 14 days or 28 days after two moxifloxacin‐containing regimens and one control regimen were analyzed using nonlinear mixed‐effects modeling. The final models were used to predict individual baseline time‐to‐positivity and time‐to‐positivity differences between 0 and 14 days or 0 and 28 days. The individual predictions served as features in the following machine learning analysis. Statistical metrics and Kaplan–Meier plots informed model selection. Bi‐exponential and exponential decay models described the 14‐day and 28‐day time‐to‐positivity data, respectively. In the machine learning analysis, baseline time‐to‐positivity and/or time‐to‐positivity differences were ranked as the most important features in all models. Statistical metrics and Kaplan–Meier plots indicated a good fit to culture conversion at 8 weeks using 4‐week phase 2a information with a C‐support vector classification model. Four‐week time‐to‐positivity phase 2a data provided more information compared to the 2‐week time‐to‐positivity data for the prediction of culture conversion. The workflow demonstrated the potential of machine learning to predict the time‐to‐event of phase 2b culture conversion up to 8 weeks using time‐to‐positivity phase 2a biomarker information in a clinical trial assessed with pharmacometric analysis.

## Study Highlights

**What Is the Current Knowledge on the Topic?:**

Phase 2a trials are typically evaluated by early bactericidal activity analysis (EBA) using time‐to‐positivity (TTP), whereas Phase 2b trials utilize culture conversion at 8 weeks as the endpoint. However, there is a challenge with current methods in linking phase 2a biomarkers with phase 2b endpoints to support phase 2b treatment design using phase 2a information in tuberculosis.

**What Question Did This Study Address?:**

This study investigates how machine learning (ML) combined with pharmacometric EBA analysis can be used to predict phase 2b culture conversion using TTP biomarker data from phase 2a trials and how different lengths of phase 2a influence the predictive ability of phase 2a biomarkers for phase 2b outcomes.

**What Does This Study Add to Our Knowledge?:**

Time‐to‐event of culture conversion in a tuberculosis phase 2b trial was well predicted using a ML classification model when phase 2a TTP biomarker, analyzed with pharmacometrics, was included. Four‐week phase 2a biomarker information improved phase 2b culture conversion predictions compared to two‐week phase 2a biomarker information.

**How Might This Change Clinical Pharmacology or Translational Science?:**

The ML combined with pharmacometrics approaches may improve regimen selection, optimize trial design, and reduce development timelines by linking phase 2a biomarkers with phase 2b endpoints. This approach could enhance early decision‐making and increase efficiency in clinical pharmacology and translational science.

## Graphical abstract

A workflow for appying pharmacometrics‐enable machine learning analysis to predict 2‐month culture conversion using phase 2a data.

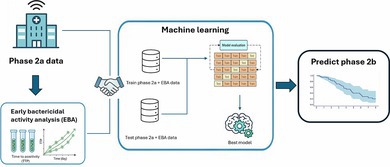

## 1 Introduction

Phase 2a studies in tuberculosis (TB) drug development often involve early bactericidal activity (EBA) [1, 2]. Early bactericidal activity analysis evaluates the decline in colony‐forming units (CFU) and/or time‐to‐positivity (TTP) in liquid culture of *Mycobacterium tuberculosis* (*M. tuberculosis*), which can provide insights into the short‐term activity of the study drug or treatment [3]. The TTP biomarker quantifies inversely the number of viable bacilli inoculated and offers advantages over CFU, which only quantifies bacterial subpopulations growing on solid media [4, 5, 6]. Pharmacometric model‐based EBA analysis is built on nonlinear mixed‐effects modeling with structural, stochastic, and covariate models [7], demonstrating significant statistical power in analyzing phase 2a data compared to traditional statistical methods [8, 9]. Phase 2b studies involve 8 weeks of treatment, with culture conversion as the primary endpoint, assessing clinical long‐term efficacy and safety. Semiparametric and parametric survival analysis techniques [10] have been used to assess treatment efficacy using culture conversion as the endpoint. Phase 2a EBA results are often used to guide phase 2b study planning, including regimen selection and safety considerations. Therefore, to bridge early findings to long‐term outcomes, it is essential to explore associations between biomarkers used in phase 2a trials and endpoints used in phase 2b trials. Machine learning (ML) has shown promising results in predicting time‐to‐event (TTE) endpoints in many different therapeutic areas, such as oncology [11], and has performed well in other survival analyses [12, 13, 14].

In this work, we investigated the utility of the phase 2a TTP biomarker data for predicting the phase 2b TTE of culture conversion in TB up to 8 weeks using ML. Additionally, we aimed to explore how the duration of phase 2a studies influences the predictability of culture conversion.

## 2 Materials and Methods

### 2.1 Clinical Data

Data from the REMoxTB trial (NCT00864383), a randomized, double‐blind, placebo‐controlled phase 3 study evaluating moxifloxacin‐containing regimens [15], was obtained from the TB‐PACTS platform (https://c‐path.org, ClinicalTrials.gov number, NCT00864383). The ethics committee at University College London and all national and local ethics committees approved the study. The US Food and Drug Administration, the Federal Institute for Drugs and Medical Devices (Bundesinstitut für Arzneimittel und Medizinprodukte), and the national regulatory authorities of the countries in which the trial was conducted reviewed and approved the protocol. All patients provided written or witnessed oral informed consent. The reason for using the REMoxTB dataset is that this dataset included 1820 patients and long‐term biomarker observations, which supports this study to explore the different scenarios for phase 2.

The study evaluated the replacement of ethambutol or isoniazid with moxifloxacin for the treatment of uncomplicated, smear‐positive pulmonary TB. The control regimen was isoniazid, rifampicin, pyrazinamide, and ethambutol for 8 weeks, followed by 18 weeks of isoniazid and rifampicin. The isoniazid group received a similar treatment as the control regimen by replacing ethambutol with moxifloxacin for 17 weeks, followed by 9 weeks of placebo, whereas patients in the ethambutol group were treated with a regimen in which isoniazid was replaced with moxifloxacin for 17 weeks, followed by 9 weeks of placebo. Only data up to 8 weeks were included in the analysis.

The time‐to‐event endpoint at week 8 was culture conversion and was defined as two consecutive negative culture results. The date of culture‐negative status was defined as the date of the first negative culture result and was maintained unless confirmed culture reversion occurred (two positive cultures without an intervening negative culture, or a single positive culture not followed by two negative cultures), as in the original analysis of the REMoxTB trial [16], using the per‐protocol study population. Further details of the data preprocessing steps are provided in Appendix S1. In the original REMoxTB clinical dataset, censoring occurred when participants withdrew from the study or had missing culture results before the end of follow‐up. For the machine learning analysis, participants without any culture observations during the predefined test period were excluded because a definitive binary outcome could not be assigned within the prediction window (Scenario B: *n* = 81; Scenario C: *n* = 113; Scenario D: *n* = 114). To address missing values, we implemented different approaches based on the type of data. Missing weight and height records for specific time points were imputed using information from the closest available recorded day. Missing categorical lung cavity data were replaced by the most frequently observed category. For missing numeric baseline TTP data, we utilized the values from available screening phase records. If screening data were also unavailable, the remaining missing entries were replaced with the global mean of the baseline TTP distribution.

### 2.2 Pharmacometric Early Bactericidal Activity Analysis

**FIGURE 1:**
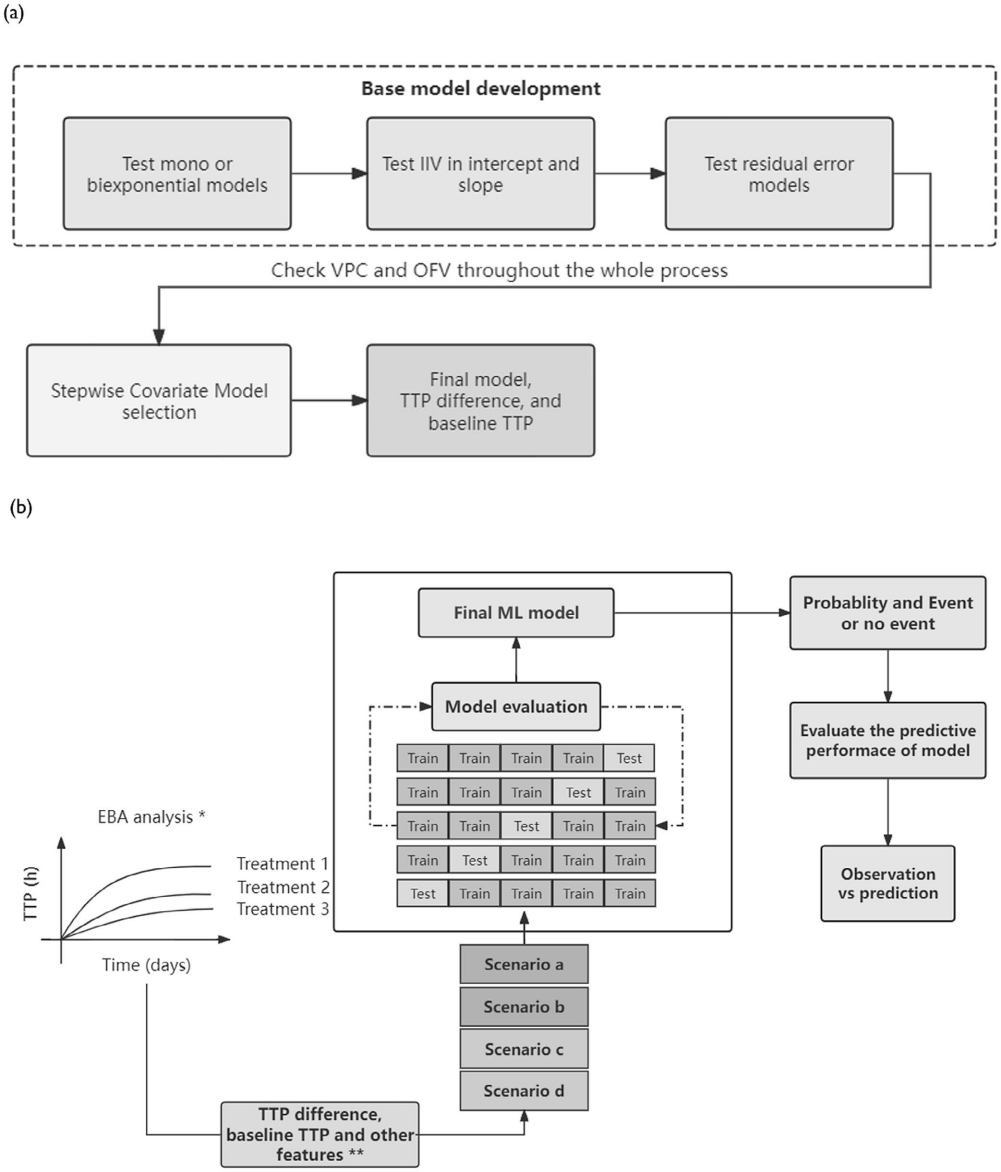
Detailed workflow for predicting culture conversion with machine learning (ML) models combined with pharmacometric model‐based early bactericidal activity (EBA) analysis. (a) EBA analysis workflow (b) detailed workflow for ML model building and evaluation *EBA analysis: Pharmacometrics early bactericidal activity analysis **Other features: Height, body weight, regimen, cavity, smoking, treatment weeks, baseline TTP. IIV, inter‐individual variability; OFV, objective function value; TTP, time to culture positivity; VPC, visual predictive check.

The approach to combine ML with pharmacometrics EBA analysis to predict culture conversion at 8 weeks involved different steps as outlined in Figure 1; pharmacometric model‐based EBA analysis, ML data splitting, ML model training and validation, prediction of culture conversion, and evaluation of ML model predictive performance.

The first step with EBA analysis involved nonlinear mixed‐effects modeling to develop two separate models, including data up to 14 days and 28 days, respectively. The former model was used to predict the individual change in TTP from day 0 to day 14 (∆TTP_0–14_), and the latter model was used to predict the individual TTP difference from day 0 to day 28 (∆TTP_0–28_). Different monoexponential Equation (1), biexponential (Equation 2), and exponential decay functions (Equation 3) were explored, such as:

(1) $$\mathrm{TTP}=e^{A_{\mathrm{bi}}}\cdot e^{-\alpha_{\mathrm{bi}}\cdot t}$$

(2) $$\mathrm{TTP}=e^{A_{\mathrm{bi}}}\cdot e^{-\alpha_{\mathrm{bi}}\cdot t}+e^{B_{\mathrm{bi}}}\cdot e^{-\beta_{\mathrm{bi}}\cdot t}$$

where $A_{\mathrm{bi}}$ is the baseline and $B_{\mathrm{bi}}$ represents the intercept for the second slope, $\alpha_{\mathrm{bi}}$ and $\beta_{\mathrm{bi}}$ are the slopes of the curve and t is the time in days.

(3) $$\mathrm{TTP}=A_{\exp}+B_{\exp}\cdot \left(1-e^{\alpha_{\exp}\cdot t}\right)$$

where $A_{\exp}$ is the intercept and $B_{\exp}$ represent the asymptote, $\alpha_{\exp}$ are the slopes of the curve and t is the time in days.

Interindividual variability (IIV) was evaluated in all model parameters. Residual variability was explored with an additive, proportional, or combined additive and proportional error modeling. A stepwise covariate modeling (SCM) was applied to evaluate body weight, age, race, sex, smoking, and cavity as covariates on slope(s), intercept, and/or asymptote. Baseline TTP was evaluated on slope(s). The difference between the treatment arms was explored as the treatment effect on slope(s).

### 2.3 Pharmacometric Model Evaluation and Selection

The models were evaluated using visual predictive checks (VPC) [17], relative standard errors (RSE) of parameter estimates, and objective function values (OFV). Selection between nested models was based on an ∆OFV ≥ 3.84 and a 5% significance level for one degree of freedom. Covariate analysis included an inclusion criterion of *p* ≤ 0.05, followed by a backward deletion criterion of *p* ≤ 0.01.

From the final EBA models, individual TTP at days 0, 14, and 28 was predicted. ∆TTP_0–14 days_ and ∆TTP_0–28 days_ were derived and used as explanatory variables (features) in the subsequent ML modeling.

### 2.4 Machine Learning Model Development

The time‐to‐event endpoint at week 8 was culture conversion and was defined as in the original analysis of the REMoxTB trial [15]. Different scenarios were defined to resemble one with rich data representing a phase 2b study and scenarios reflecting different phase 2a study lengths, as shown in Table S1. In scenario A, 80% of the phase 2b culture conversion dataset was used for ML model training, and 20% was used for the prediction of culture conversion at 8 weeks (56 days). In scenario B, culture conversion up to 2 weeks was used to train the model to predict culture conversion at 8 weeks. In scenario C, culture conversion up to 4 weeks was used to train the model to predict culture conversion at 8 weeks, and in scenario D, culture conversion data up to 2 weeks of treatment were used to train the model to predict culture conversion at 4 weeks.

In the model training phase, different training datasets (scenarios A–D) were modeled using four different ML models: extreme gradient boosting (XGBoost), random Forest (RF), C‐support vector classification (SVC), and k‐nearest neighbors algorithm (KNN). Bayesian optimization was applied to fine‐tune the hyperparameters of each model. To improve model generalization and reliability, we applied five‐fold cross‐validation. The training dataset was partitioned into five subsets (folds) while maintaining the baseline prevalence of the culture conversion outcome in each fold. In each of the five iterations, four folds were used for model training, while the remaining fold served as the internal validation set. This process was rotated so that each data point was used for both training and validation exactly once. The final model performance metrics were reported across all five folds to support the final model selection. Different techniques were explored to improve model performance and address class imbalance. We employed the synthetic minority over‐sampling technique (SMOTE) on the training dataset to oversample the minority class. To optimize the culture conversion decision threshold, we implemented a nested validation strategy. The training set was split into a sub‐training set and a validation set. We performed a grid search for the optimal threshold (ranging from 0 to 1) by maximizing prediction accuracy to balance precision and recall under imbalanced class distributions. This optimized threshold was derived using the subtraining data, confirmed on the validation set to prevent overfitting, and applied to the test set for final performance evaluation. We also defined a loss function by giving weights to different evaluation metrics (Equation 4).

(4) $$\begin{array}{c} \text{Loss score}=0.7\times \text{Mean}\;\mathrm{AUC}+0.3\times \text{Mean recall} \end{array}$$

Different variables were investigated as predictors for scenarios B, C, and D in the ML models, such as height, body weight, drug regimen, lung cavity, smoking, treatment day, baseline TTP, and ∆TTP_0–14 days_ or ∆TTP_0–28 days_. For scenario A, height, body weight, drug regimen, lung cavity, smoking, and treatment day were included. Missing individual information on height and body weight on a specific day was replaced using the closest available records, while categorical missing values for lung cavity were replaced with the most common category.

Based on the final ML models, the probability of the event occurring from each model was predicted, and individual predictions of culture conversion at 8 weeks or censoring were derived.

### 2.5 Machine Learning Model Selection and Evaluation

In the model evaluation phase (Figure 1), the predictive performance of each ML model was evaluated using the area under the receiver operating characteristic curve (ROC‐AUC), the concordance index (C‐index) metrics, and Kaplan–Meier (KM) plots to guide model selection. The ROC‐AUC measures how well the model distinguishes between positive events and negative events, i.e., censoring, where a higher AUC indicates better overall model performance. The C‐index represents the discriminatory power of survival models, showing how well the model ranks survival times. A higher C‐index reflects a more accurate ranking of event times. We applied 1000 bootstraps to calculate confidence intervals (CIs) for the ROC‐AUC and C‐index.

### 2.6 Softwares

The EBA analysis was performed using NONMEM (version 7.5.1, ICON Development Solutions, Hanover, MD, USA) [16] on a Unix cluster using the GNU Fortran compiler (version 5.1.0) through the PsN toolkit (version 7.5.0, Department of Pharmaceutical Biosciences, Uppsala University, Uppsala, Sweden) [18]. The ML analysis with data preparation, model building, and evaluation metrics was done using Python (version 3.7) [19]. The Kaplan–Meier plots were created using R (version 4.1.0) [20]. More details of the environment and model code can be found in the Supplementary file.

## 3 Results

### 3.1 Clinical Data

**FIGURE 2:**
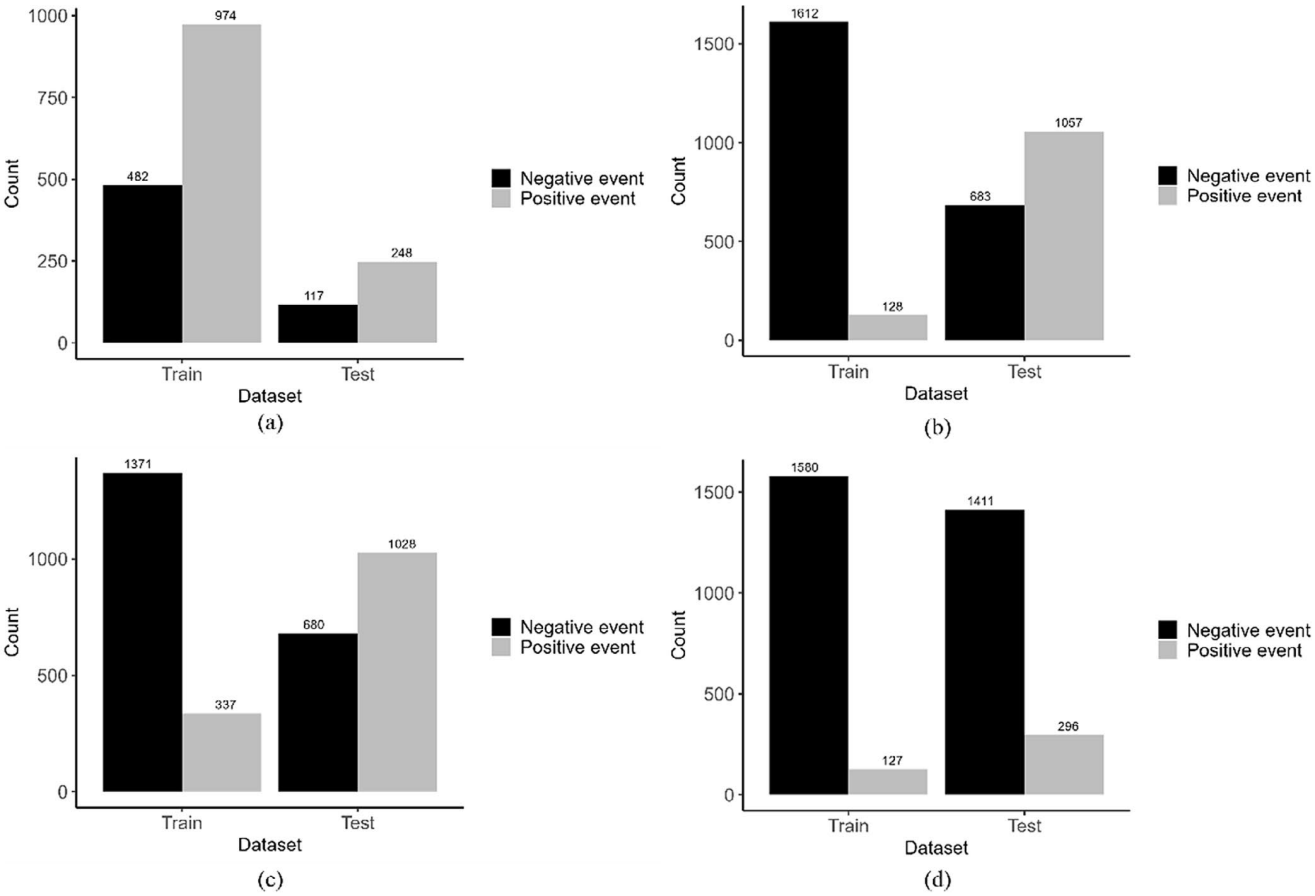
Distribution of training data and prediction data for different scenarios; (a) scenario A: An 80% training and 20% testing split of the entire dataset; (b) scenario B: Training on up to 2 weeks of data for testing on up to 56 days; (c) scenario C: Training on up to 4 weeks for testing on up to 56 days; (d) scenario D: Training on up to 2 weeks for testing on up to 4 weeks; Blue line is the observation event; Pink area is the 90% prediction interval.

The analysis dataset was divided into different scenarios (Table S1). In total, scenario A included 1821 patients, 1740 patients were included in scenario B, 1708 patients in scenario C, and 1707 patients in scenario D. The culture conversion event distribution across the different scenarios is shown in Figure 2. In scenarios A and D, the training and prediction datasets had similar event distributions. However, scenarios B and C had data imbalance issues. In scenario B, the training set had fewer positive events (culture conversion) compared to negative events (censoring). The scenario C training dataset included more positive events than scenario B, but it still didn't match the distribution in the prediction set.

### 3.2 Pharmacometric Early Bactericidal Activity Analysis

**FIGURE 3:**
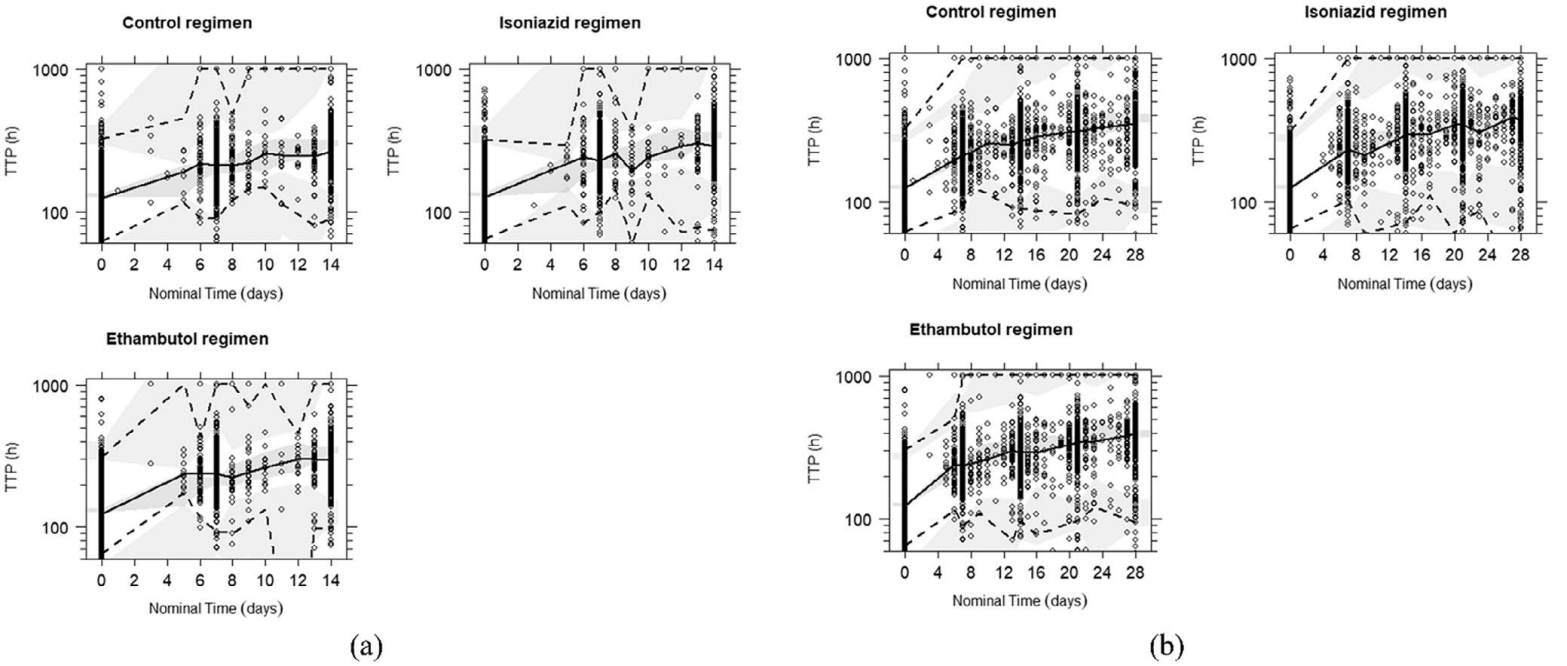
Visual predictive check (VPC) of early bactericidal activity using time‐to‐positivity (TTP) (a) using the final model with TTP data up to 2 weeks and (b) using the final model with TTP data up to 4 weeks. Prediction‐corrected observations are presented as empty circles, the median is shown as a solid line, and 2.5th and 97.5th percentiles are shown as dashed lines. Dark shaded area represents a 95% confidence interval for the median, and the light shaded areas represent the 95% confidence intervals for the 2.5th and 97.5th percentiles of data based on 1000 simulations.

The TTP_0–14 days_ was best described using a biexponential model, and the VPC indicated a good fit of the final model to the observed data (Figure 3). Cavity, sex, and smoking were significant covariates on the TTP baseline, while race, sex, age, and smoking were identified on the slopes and the intercept. The final model included a proportional error model and IIV in the baseline and slopes. The median predicted baseline TTP and ∆TTP_0–14 days_ were 126.8 and 182 h. The shrinkage in IIV was 27% (baseline) and 47% (slope), while the shrinkage in proportional residual error was 14%. Final parameter estimates are shown in Table S2.

The TTP_0–28 days_ followed an exponential decay model, with the VPC confirming a good fit (Figure 3). Cavity, age, sex, weight, and race were significant covariates on the asymptote. Cavity and smoking were identified on the intercept, while baseline TTP, sex, and race were identified on the slope. The final model included a proportional error model and IIV in the intercept, slope, and asymptote. The median predicted baseline TTP and ∆TTP_0–28 days_ were 126.8 and 240.3 h. The shrinkage was 18% (intercept), 44% (slope), and 31% (asymptote) for IIV, and 12% for residual error. Final parameter estimates are shown in Table S3.

No statistically significant difference between the three treatments was found for both EBA models.

### 3.3 Final ML Models and Predictive Performance

**FIGURE 4:**
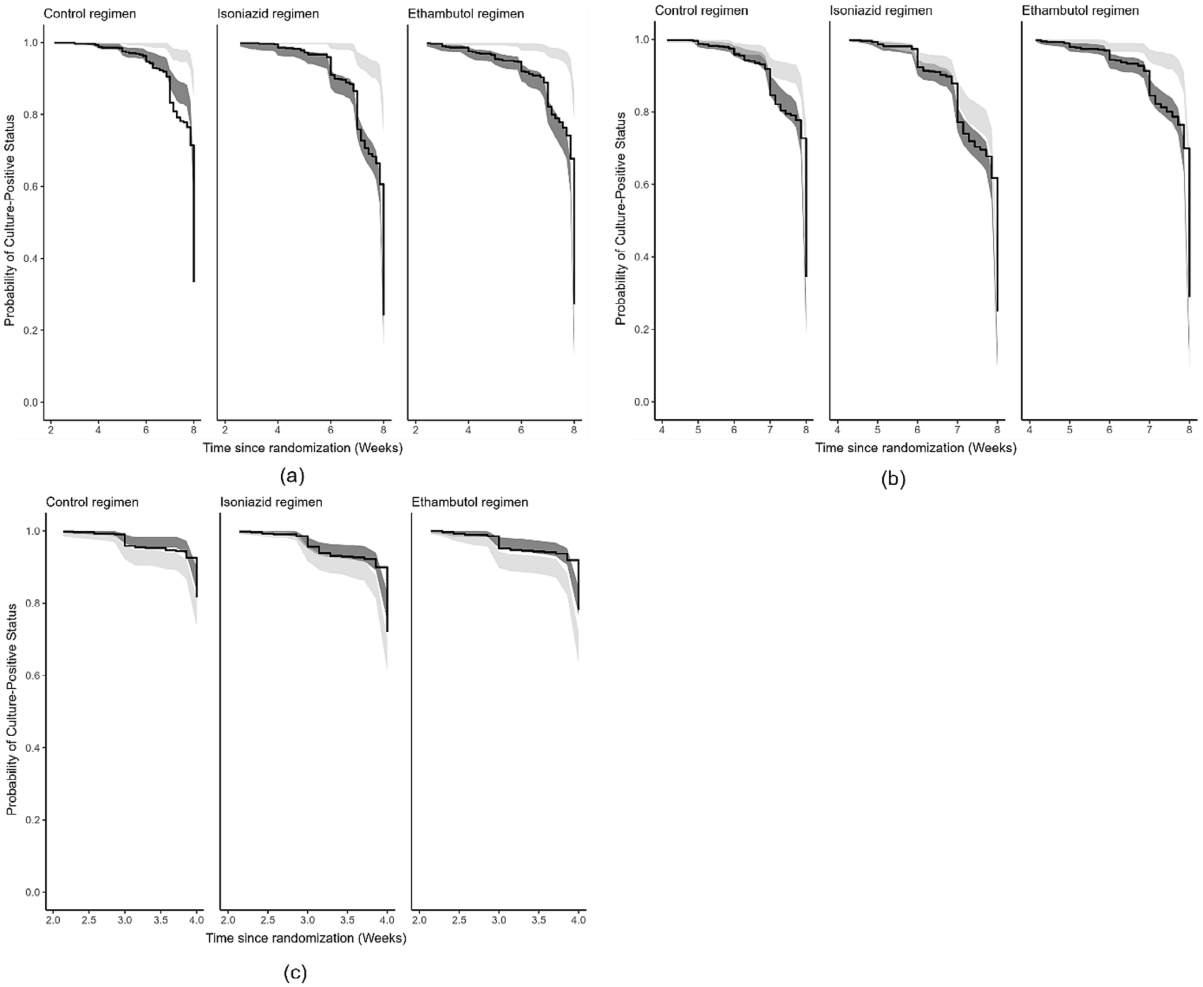
Kaplan Meier plot for prediction of culture conversion with EBA and without EBA in (a) scenario B: Training on up to 2 weeks of data for testing on up to 56 days; (b) scenario C: Training on up to 4 weeks for testing on up to 56 days; (c) scenario D: Training on up to 2 weeks for testing on up to 4 weeks. The dark gray area is the 90% prediction confidence interval from the model combined with EBA. The light gray area is the 90% prediction confidence interval from the model combined without EBA. The black line is the observed data.

Adjusting the decision thresholds of classifiers helped improve model performance in handling the data imbalance, but introducing baseline TTP, ∆TTP_0–14 days_ and ∆TTP_0–28 days_ as explanatory variables also enhanced the models' predictive performance (Figure 4). In the end, including explanatory variables in the ML models was chosen instead of adjusting the thresholds of classifiers, as this simplified the models and resulted in better ROC‐AUC and C‐index values. Using both adjusted decision thresholds together with explanatory variables was not better than only using explanatory variables in the ML models.

**FIGURE 5:**
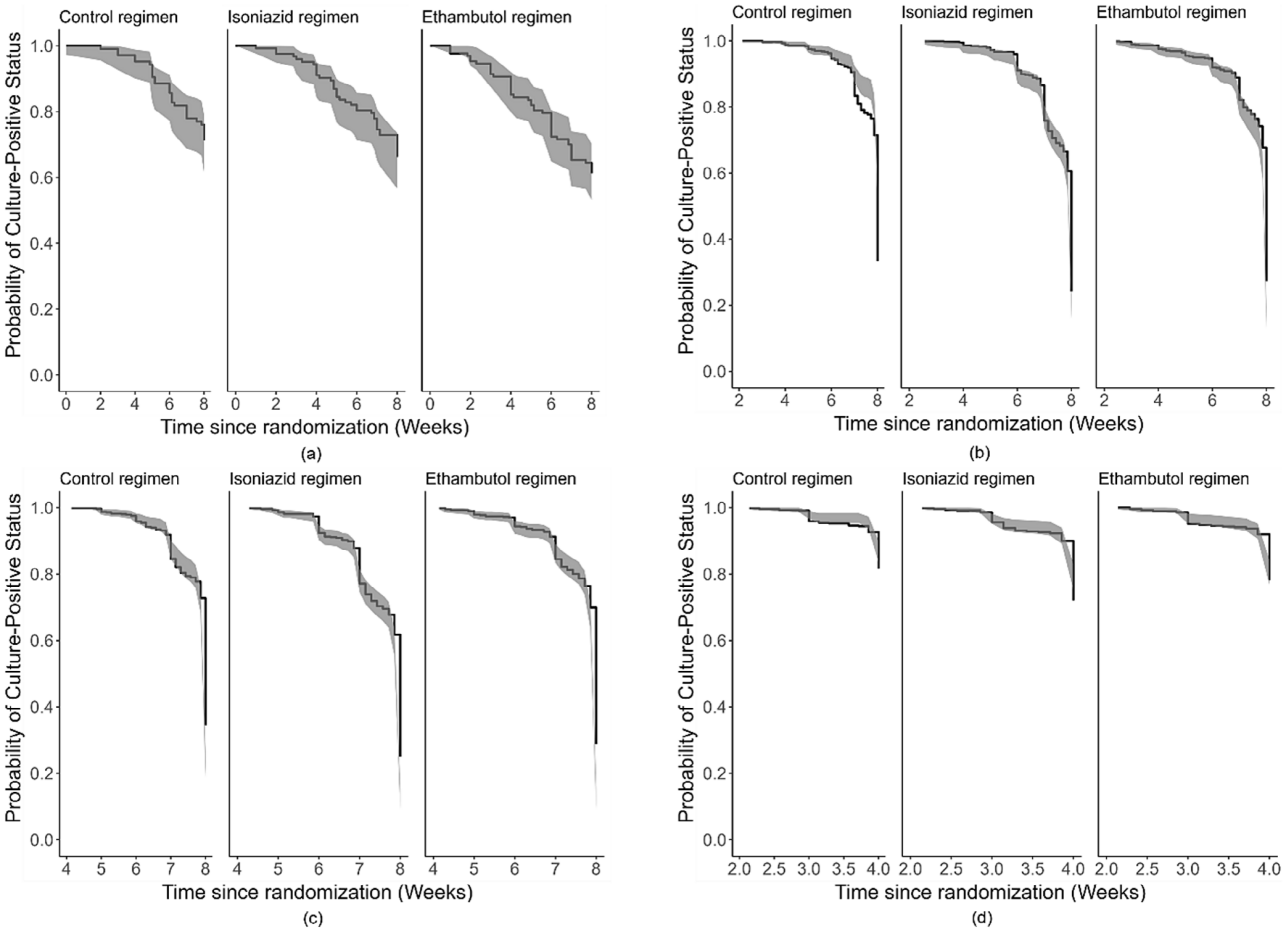
Kaplan Meier plot for prediction of culture conversion in (a) scenario A: An 80% training and 20% prediction split of the entire dataset; (b) scenario B: Training on up to 2 weeks of data for testing on up to 56 days; (c) scenario C: Training on up to 4 weeks for testing on up to 56 days; (d) scenario D: Training on up to 2 weeks for testing on up to 4 weeks. The black line is the observed data, and the gray area is the 90% prediction confidence interval from the model.

The model performance was evaluated by statistical metrics as shown in Table 1. In Scenario A, using the whole phase 2b dataset with an 80% training and 20% prediction split, the XGBoost model achieved the highest ROC‐AUC score of 0.930 (95% CI: 0.894–0.959), while the SVC model yielded the highest C‐index of 0.793 (95% CI: 0.753–0.829), indicating strong predictive performance. Scenario B involved training on data up to 2 weeks and testing on data up to 56 days. This resulted in lower predictive performance compared to Scenario A, with XGBoost leading the performance with an ROC‐AUC of 0.700 (95% CI: 0.675–0.725) and a C‐index of 0.700 (95% CI: 0.675–0.726). Scenario C focused on training on data for up to 4 weeks and testing on data for up to 56 days. Performance improved over Scenario B, where the RF model demonstrated the highest ROC‐AUC of 0.762 (95% CI: 0.739–0.787) and a C‐index of 0.651 (95% CI: 0.631–0.671). Scenario D consisted of training on data for up to 2 weeks and testing on data for up to 4 weeks. In this scenario, SVC led the performance with an ROC‐AUC of 0.763 (95% CI: 0.731–0.795) and a C‐index of 0.720 (95% CI: 0.684–0.757). Overall, the final ML models for scenarios A, C, and D demonstrated a good fit to the culture conversion data. As shown in Figure 5, the KM plots stratified by regimen indicate that the observed survival from the data generally remained within the 90% confidence interval of the model predictions, highlighting reliable alignment of the model predictions with observed outcomes. In contrast, scenario B could only accurately predict TTE data up to 6 weeks. However, from weeks 6 to 8, the model overestimated the event probability, suggesting a reduced predictive accuracy in the later weeks for this scenario.

**TABLE 1:** Statistical metrics with 95% confidence interval for prediction of phase 2b culture conversion in scenarios A–D using different amounts of phase 2a biomarker information with the different machine learning models extreme gradient boosting (Xgboost), C‐support vector classification (SVC), Random Forest (RF) and K‐nearest neighbors algorithm (KNN).

	Scenario A		Scenario B		Scenario C		Scenario D	
	ROC‐AUC (95% CI)	C‐index (95% CI)	ROC‐AUC (95% CI)	C‐index (95% CI)	ROC‐AUC (95% CI)	C‐index (95% CI)	ROC‐AUC (95% CI)	C‐index (95% CI)
Xgboost	0.930 (0.894–0.959)	0.728 (0.687–0.768)	0.700 (0.675–0.725)	0.700 (0.675–0.726)	0.706 (0.681–0.730)	0.626 (0.605–0.647)	0.676 (0.637–0.711)	0.658 (0.622–0.694)
SVC	0.925 (0.893–0.954)	0.793 (0.753–0.829)	0.566 (0.539–0.592)	0.393 (0.368–0.418)	0.663 (0.637–0.688)	0.486 (0.455–0.517)	0.763 (0.731–0.795)	0.720 (0.684–0.757)
RF	0.929 (0.894–0.957)	0.736 (0.697–0.773)	0.668 (0.643–0.693)	0.601 (0.580–0.620)	0.762 (0.739–0.787)	0.651 (0.631–0.671)	0.684 (0.644–0.720)	0.658 (0.623–0.698)
KNN	0.924 (0.887–0.955)	0.712 (0.670–0.753)	0.640 (0.617–0.664)	0.578 (0.558–0.598)	0.687 (0.662–0.714)	0.599 (0.576–0.620)	0.655 (0.625–0.684)	0.650 (0.625–0.677)

Abbreviations: C‐index, the concordance index calculated on the independent test set; CI, 95% confidence interval estimated using 1000 bootstrap resampling; ROC‐AUC, Receiver‐operating characteristic curve—Area under the curve calculated on the independent test set.

**FIGURE 6:**
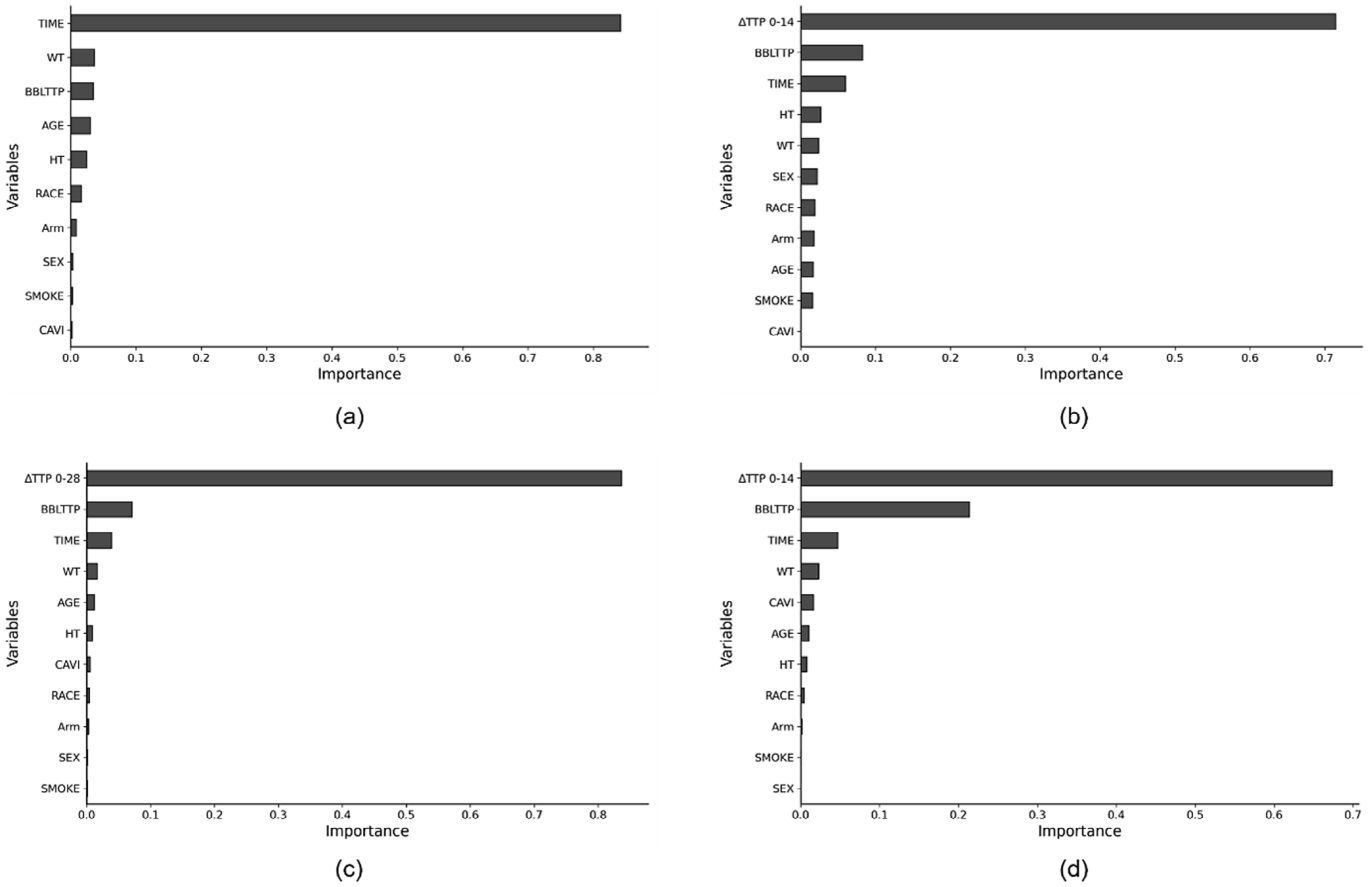
Feature selection in the final model for each scenario: (a) scenario A: An 80% training and 20% prediction split of the entire dataset; (b) scenario B: Training on up to 2 weeks of data for testing on up to 56 days; (c) scenario C: Training on up to 4 weeks for testing on up to 56 days; (d) scenario D: Training on up to 2 weeks for testing on up to 4 weeks.

Figure 6 illustrates the selection of explanatory variables importance based on the final models for each scenario. In all scenarios, baseline TTP and/or TTP differences were identified as key explanatory variables. Beyond baseline TTP and/or TTP differences, scenarios A, C, and D recognized time since the start of treatment and body weight as important explanatory variables. In scenario B, height was selected as the important explanatory variable, while age was identified in scenario A.

**FIGURE 7:**
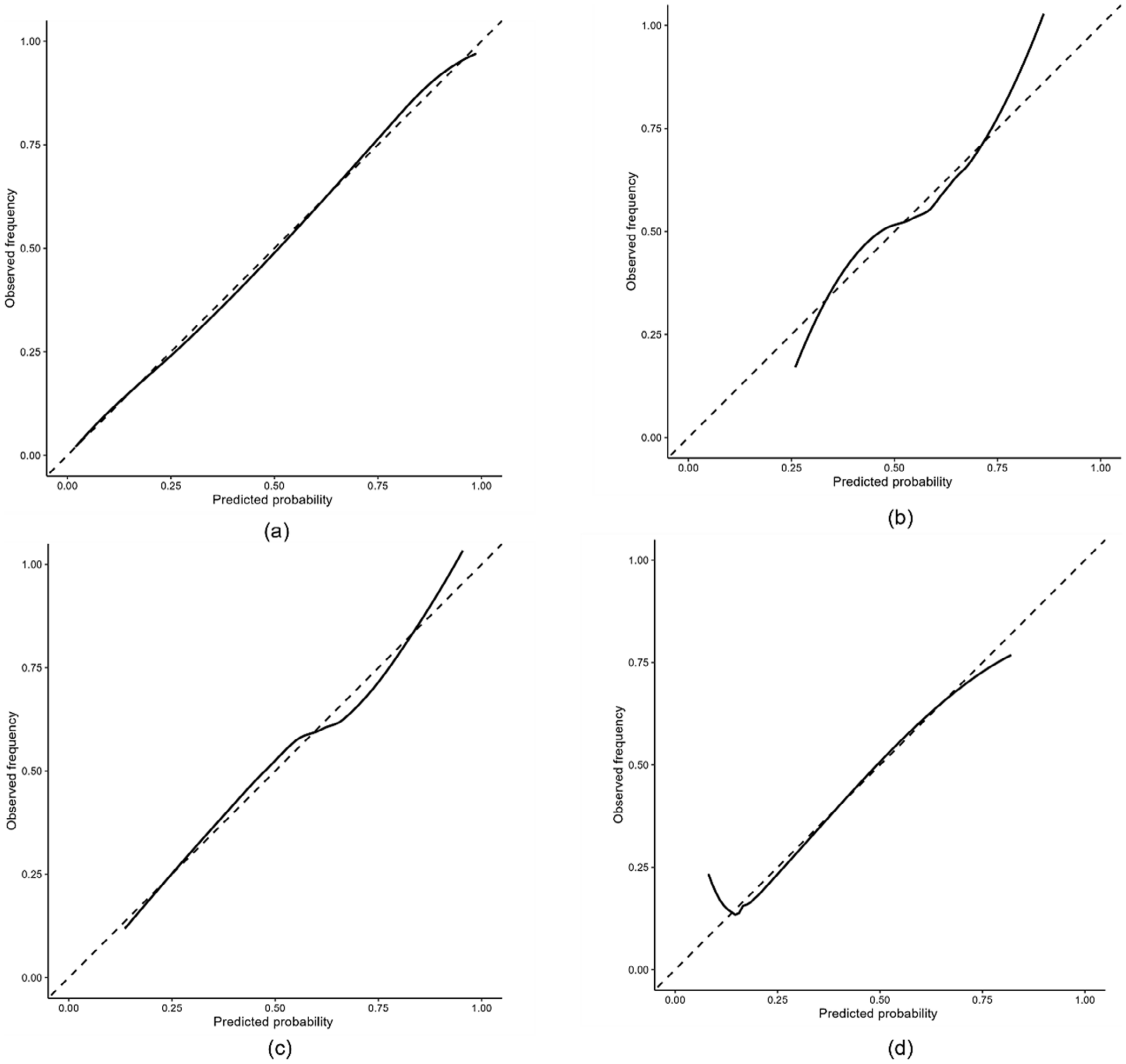
Calibration plot for best model prediction of culture conversion in (a) scenario A: An 80% training and 20% prediction split of the entire dataset; (b) scenario B: Training on up to 2 weeks of data for testing on up to 56 days; (c) scenario C: Training on up to 4 weeks for testing on up to 56 days; (d) scenario D: Training on up to 2 weeks for testing on up to 4 weeks. The black line is the observed data, and the gray area is the 90% prediction confidence interval from the model.

Calibration metrics were further applied to evaluate the reliability of the predicted probabilities for the best model identified by both statistical metrics and the KM plots in each scenario (Table 2 and Figure 7). In scenario A, the XGBoost model demonstrated the strongest calibration, with the lowest Brier score (0.080) and a low ECE (0.029), as reflected by the calibration curve closely following the diagonal identity line (Figure 7a). Scenario B exhibited the highest Brier score (0.227) and log loss (0.646), indicating greater uncertainty in predictions when using limited early data. In scenario C, despite a moderate Brier score of 0.208, the RF model achieved the lowest ECE (0.026) among all scenarios. Scenario D maintained consistent calibration results with a Brier score of 0.139 and an ECE of 0.028. As shown in Figure 7, the calibration curves for scenarios C and D remained generally well aligned with observed frequencies, confirming that the ML combined with the EBA workflow provides reliable probability estimates.

**TABLE 2:** Calibration metrics for the best model in the prediction of phase 2b culture conversion in scenarios A–D using different amounts of phase 2a biomarker information.

Scenario	Model	Brier	Log‐loss	ECE
A	XGBoost	0.080	0.282	0.029
B	RF	0.227	0.646	0.031
C	RF	0.208	0.601	0.026
D	SVC	0.139	0.45	0.028

Abbreviations: C‐index, the concordance index; CI, 95% confidence interval; ECE, expected calibration error; RF, random forest; ROC‐AUC, receiver‐operating characteristic curve—area under the curve; SVC, C‐support vector classification; XGBoost, extreme gradient boosting.

## 4 Discussion

This study applied ML models to predict culture conversion using 2 weeks, 4 weeks, and 8 weeks of TTP biomarker data. By evaluating the predictive performance of different ML models using differing amounts of training data, this study demonstrates the potential of ML models to use phase 2a biomarker data for predicting phase 2b culture conversion outcomes, thereby enhancing the understanding of phase 2a–2b relationships and exploring the potential of ML in clinical trial planning.

Our analysis highlighted that scenario A, which used 80% of phase 2b data to build models, demonstrated high accuracy in predicting culture conversion outcomes in the remaining 20% of phase 2b data. This scenario reflects an ideal setting with rich data supporting the training of ML models. In contrast, scenarios B, C, and D presented more challenging scenarios, but more closely resembled reality, as predicting culture conversion up to 4 weeks or 56 days based on 2 weeks or 4 weeks phase 2a data is more applicable for phase 2b planning in drug development. However, this scenario had data imbalance due to time‐based data splitting, with fewer positive events in the training dataset but more in the prediction dataset (Figure 2). This imbalance makes it harder for models to learn effectively due to insufficient positive events in the training phase. This was handled in the analysis by introducing baseline TTP, ∆TTP_0–14 days_ and ∆TTP_0–28 days_ as informative explanatory variables, which resulted in good predictions for all scenarios.

Prediction of long‐term culture conversion outcomes based on early phase 2a data is crucial for planning phase 2b trials. Accurate predictions can guide treatment duration, evaluate potential drug efficacy, and modify trial designs without waiting for complete data from phase 2b. Specifically, scenario C, which used data from the first four weeks to train the model, showed improved accuracy compared with scenario B because more patients achieved culture conversion after two weeks, providing more informative data than using only two‐week data (Figure 4). This suggests that using an extended phase 2a study period, i.e., 4 weeks, captures more relevant information and enhances predictive performance compared with using only two‐week EBA studies. Similarly, other research has shown that using TTP data up to 6 weeks from the start of treatment can improve the predictive performance for predicting phase 2b outcomes [21].

The results show that incorporating predictions from the pharmacometric EBA biomarker models of baseline TTP, ∆TTP_0–14 days_ and ∆TTP_0–28 days_ as additional explanatory variables in the ML models significantly enhanced the performance of the ML models, showing the ability of phase 2a biomarker information to predict phase 2b outcomes. In addition, inclusion of predicted TTP baseline and differences instead of observed TTP improved the ML models' performance, as predictions from pharmacometrics EBA modeling do not include measurement errors and other unknown sources of errors from the observed TTP. Baseline TTP has earlier been shown to be correlated with treatment success [22]. Although Gillespie et al. (2014) [15] demonstrated a more rapid initial decline in bacterial load in the moxifloxacin‐containing regimens, the present study focused on predicting the clinical event rather than describing longitudinal TTP trajectories. The treatment effect on early bacterial clearance was incorporated into the ML models through pharmacometrically derived variables (baseline TTP, ΔTTP_0–14_, and ΔTTP_0–28_). These variables capture both regimen‐specific effects and individual response variability. Therefore, the categorical treatment arm was not selected as the most important covariate because it did not provide additional predictive value since this information from EBA already informed these predictors.

In our EBA modeling, we observed a parameter shrinkage of up to 47% for the slope. Such high shrinkage suggests that individual‐level data were insufficiently informative to allow specific parameters to deviate significantly from the population mean, potentially hindering the ML models' ability to discern fine‐grained individual signals. However, our workflow demonstrates the advantage of ML in feature integration. By considering the full spectrum of covariates, the ML models maintained robust predictive performance, compensating for the statistical uncertainty associated with individual EBA estimates.

This work demonstrates the potential of ML models to maintain predictive accuracy even in imbalanced datasets, especially when informative variables like baseline TTP, ∆TTP_0–14 days_ and ∆TTP_0–28 days_ are included. This property is particularly valuable in clinical trials, where diverse patient populations or incomplete data often lead to similar challenges. Furthermore, all models identified baseline TTP and/or TTP differences as important variables, which is consistent with previous research exploring their role in understanding bacterial load dynamics for predicting culture conversion [23]. Interestingly, the models did not identify the treatment arm as an important variable, aligning with the original research findings that showed no statistically significant differences among regimens [15].

Machine learning can predict outcomes without requiring a prior understanding of complex data structures or biological mechanisms [24], which leads to highly effective and efficient clinical data analysis. However, the strengths of ML can become limitations in certain scenarios. When the dataset is not sufficiently informative, it may struggle to make accurate predictions [25]. In such cases, mechanistic quantitative systems pharmacology (QSP) models can incorporate expert knowledge and provide more reliable insights. But at the same time, the mechanistic models can also become incredibly complicated with the number of known and unknown parameters [26].

An ideal approach would combine mechanistic QSP models with ML to benefit from the strengths of both [27]. Mechanistic models could serve as intermediate inputs and a validating framework, ensuring predictions align with known principles [28]. Meanwhile, ML could handle more complex datasets and scale to broader applications, overcoming the limitations of mechanistic models in diverse or high‐dimensional data environments.

This study demonstrates the potential of ML models to use short‐term data to predict the long‐term efficacy. Furthermore, this tool is straightforward to use, making it accessible in clinical settings. In the future, incorporating biological mechanisms into ML models and linking QSP to ML models could further enhance the understanding of treatment outcomes.

The primary limitation of this study is that this study it is based only on data from the REMoxTB trial to establish our ML models, which was not a dedicated phase 2a trial and may introduce discrepancies in data collection procedures compared with typical phase 2a trials. Future work should aim to incorporate data from a broader range of trials to validate and refine our approach of integrating the pharmacometric EBA model with ML in predicting 2‐month culture conversion using phase 2a data.

Another limitation of this study is that models currently include only one biomarker, i.e., TTP, for analysis. Expanding the model to incorporate further biomarkers, such as the most probable number (MPN) [29], molecular bacterial load assay (MBLA) [30], R/S ratio [31] or LAM [32] could provide a more comprehensive understanding of drug efficacy and treatment outcome.

Additionally, this study excluded participants without culture observations during the test period from the machine learning analysis. Because the machine learning endpoint required an evaluable culture conversion outcome within the prediction window, these participants could not be assigned a definitive binary outcome and therefore could not contribute to model training or evaluation. Although the proportion of excluded participants was small, this approach may limit the generalizability of the findings and could introduce selection bias if excluded participants differ systematically from those included. Future studies should investigate strategies to minimize missing outcome data and evaluate the robustness of prediction models in more populations. In conclusion, ML models combined with phase 2a pharmacometric EBA show potential as a valuable tool for predicting phase 2b outcomes based on phase 2a information. The inclusion of phase 2a TTP information predicted phase 2b outcomes, demonstrating even greater performance when an extended phase 2a trial duration of 4 weeks was employed to forecast phase 2b culture conversion. This study underlines the potential of ML in enhancing the predictive accuracy of culture conversion and supporting the planning of future clinical trials.

## Author Contributions

All authors wrote the manuscript, designed the research, and performed the research. H.Y. analyzed the data.

## Funding

This work was supported by the Innovative Medicines Initiative 2 Joint Undertaking (JU) under grant agreement No 101007873. The JU receives support from the European Union's Horizon 2020 research and innovation programme and EFPIA, Deutsches Zentrum für Infektionsforschung e. V. (DZIF), and Ludwig‐Maximilians‐Universität München (LMU). EFPIA/AP contribute to 50% of funding, whereas the contribution of DZIF and the LMU University Hospital Munich has been granted by the German Federal Ministry of Education and Research. USHS was the principal investigator and achiever of the grant.

## Conflicts of Interest

The authors declare no conflicts of interest.

## Supporting information


**Appendix S1:** Supporting Information.

**Table S1:** Scenarios for different data‐splitting strategies for prediction of phase 2b 2‐month culture conversion using machine learning with pharmacometric model‐based phase 2a time‐to‐positivity (TTP).
**Table S2:** Final parameter estimates of the early bactericidal activity model with time‐to‐positivity from days 0 to 14 days using pharmacometric nonlinear mixed‐effects modeling.
**Table S3:** Final parameter estimates of the early bactericidal activity model with time‐to‐positivity from days 0 to 28 days using pharmacometric nonlinear mixed effects modeling.
