# Supplementary material for: Applying a Pharmacometrics‐Enabled Machine Learning Analysis to Predict 2‐Month Culture Conversion Using Phase 2a Data in a Tuberculosis Clinical Trial
Source: Clin Transl Sci. 2026 Aug 10;19(8):e70694. doi: 10.1111/cts.70694 (PMC13457353; doi:10.1111/cts.70694)
Supplement: Supplementary file 1 [file CTS-19-e70694-s002.docx]

Supplementary Information: Applying a pharmacometrics-enabled machine learning analysis to predict 2-month culture conversion using Phase 2a data in a tuberculosis clinical trial

*By Huifang You^1^, Ulrika S.H. Simonsson*

1 *Department of Pharmaceutical Biosciences, Uppsala University, Uppsala, Sweden*

**TABLE S1.**  Scenarios for different data splitting strategies for prediction of phase 2b 2-month culture conversion using machine learning with pharmacometric model-based phase 2a time-to-positivity (TTP)

| **Scenario** | **Training data** | **Prediction** |
| --- | --- | --- |
| A | 80 % of all data | 20% of all data (culture conversion at any time point) |
| B | TTP and culture conversion data up to week 2 | culture conversion up to 2 months |
| C | TTP and culture conversion data up to week 4 | culture conversion up to 2 months |
| D | TTP and culture conversion data up to week 2 | culture conversion up to 4 weeks |

**TABLE S2.** Final parameter estimates of the early bactericidal activity model with time-to-positivity from days 0 to 14 days using pharmacometric nonlinear mixed effects modelling

| **Parameter** | **Estimate** | **RSE (%)** | **Shrinkage (%)** |
| --- | --- | --- | --- |
| $A_{bi}$ | 3.43 | 2 | - |
| $\alpha_{bi}$_isoniazid or ethambutol_ | 0.0477 | 6 | - |
| $\alpha_{bi}$_control_ | 0.0419 | 8.3 | - |
| $B_{bi}$_isoniazid or ethambutol_ | 4.56 | 0.6 | - |
| $B_{bi}$_control_ | 4.54 | 0.7 | - |
| $\beta_{bi}$_isoniazid or ethambutol_ | 0.0737 | 2 | - |
| $\beta_{bi}$_control_ | 0.0645 | 2.8 | - |
| Absence of cavity on $A_{bi}$ | 0.124 | 13.5 | - |
| Female sex on $A_{bi}$ | -0.00554 | 30.5 | - |
| Smoking on $A_{bi}$ | 0.061 | 23.8 | - |
| Asian race on $B_{bi}$ | -0.0251 | 17.6 | - |
| Unknown race on $B_{bi}$ | -0.0144 | 35.2 | - |
| Female sex on $B_{bi}$ | 0.0223 | 21.9 | - |
| Unknown race on $\beta_{bi}$ | 0.963 | 37.1 | - |
| Age on $\beta_{bi}$ | -0.00489 | 21.1 | - |
| Smoking on $\beta_{bi}v$ | -0.0867 | 28.8 | - |
| $\omega_{baseline}^{2}$ (% CV) | 0.0427 | 10.2 | 27 |
| $\omega_{slope}^{2}$(% CV) | 0.785 | 9.7 | 47 |
| $\sigma^{2}$ (% CV) | 0.0981 | 2.8 | 14 |

$A_{bi}$: baseline; $B_{bi}$: intercept; $\alpha_{bi}$: slope; $\beta_{bi}$: slope; $\omega^{2}$inter-individual variability on variance scale; $\sigma^{2}$: proportional residual error; RSE: relative standard error; CV: coefficient of variation

| **Parameter** | **Estimate** | **RSE (%)** | **Shrinkage (%)** |
| --- | --- | --- | --- |
| $A_{exp}$ | 117 | 1.1 | - |
| $\alpha_{exp}$_control_ | 0.0472 | 13.3 | - |
| $\alpha_{exp}$_isoniazid_ | 0.0441 | 13.2 | - |
| $\alpha_{exp}$_ethambutol_ | 0.0599 | 11.3 | - |
| $B_{exp}$_control_ | 341 | 8.8 | - |
| $B_{exp}$_isoniazid_ | 425 | 9.1 | - |
| $B_{exp}$_ethambutol_ | 341 | 7.2 | - |
| Age on $B_{exp}$ | -0.00662 | 17.7 | - |
| Absence of cavity on $B_{exp}$ | 0.129 | 39.2 | - |
| Asian race on $B_{exp}$ | -0.121 | 26.5 | - |
| Female sex on $B_{exp}$ | 0.401 | 34.4 | - |
| Weight on $B_{exp}$ | 0.00793 | 19.3 | - |
| Absence of cavities on $A_{exp}$ | 0.154 | 16.9 | - |
| Black race on $B_{exp}$ | 0.1 | 20.6 | - |
| Smoking on $A_{exp}$ | 0.0559 | 33.1 | - |
| Baseline TTP on $\alpha_{exp}$ | 0.00388 | 18 | - |
| Black race on $\alpha_{exp}$ | -0.184 | 29.2 | - |
| Female sex on $\alpha_{exp}$ | -0.336 | 28.6 | - |
| $\omega_{intercept}^{2}$ (%CV) | 0.0788 | 7.2 | 18 |
| $\omega_{slope}^{2}$ (%CV) | 0.34 | 14.8 | 44 |
| $\omega_{asymptote}^{2}$ (%CV) | 0.227 | 6.4 | 31 |
| $\sigma^{2}$ (%CV) | 0.104 | 2.5 | 12 |

**TABLE S3.** Final parameter estimates of the early bactericidal activity model with time-to-positivity from days 0 to 28 days using pharmacometric nonlinear mixed effects modelling.

$A_{exp}$: intercept; $B_{exp}$: asymptote; $\alpha_{exp}:$ slope; $\omega^{2}$inter-individual variability; $\sigma^{2}$: proportional residual error; RSE: relative standard error; CV: coefficient of variation
